# Detecting Mechanical Anisotropy of the Cornea Using Brillouin Microscopy

**DOI:** 10.1167/tvst.9.7.26

**Published:** 2020-06-24

**Authors:** Joshua N. Webb, Hongyuan Zhang, Abhijit Sinha Roy, James Bradley Randleman, Giuliano Scarcelli

**Affiliations:** 1 Fischell Department of Bioengineering, University of Maryland, College Park, MD, USA; 2 Cole Eye Institute, Cleveland Clinic, Cleveland, OH, USA; 3 Narayana Nethralaya Foundation, Bangalore, India

**Keywords:** Brillouin microscopy, corneal anisotropy, corneal cross-linking, keratoconus

## Abstract

**Purpose:**

The purpose of this study was to detect the mechanical anisotropy of the cornea using Brillouin microscopy along different perturbation directions.

**Methods:**

Brillouin frequency shift of both whole globes (*n* = 10) and cornea punches (*n* = 10) were measured at different angles to the incident laser, thereby probing corneal longitudinal modulus of elasticity along different directions. Frequency shift of virgin (*n* = 26) versus cross-linked corneas (*n* = 15) over a large range of hydration conditions were compared in order to differentiate the contributions to Brillouin shift due to hydration from those due to stromal tissue.

**Results:**

We detected mechanical anisotropy of corneas, with an average frequency shift increase of 53 MHz and 96 MHz when the instrument probed from 0° to 15° and 30° along the direction of the stromal fibers. Brillouin microscopy did not lose sensitivity to mechanical anisotropy up to 96% water content. We experimentally measured and theoretically modeled how mechanical changes independent of hydration affect frequency shift as a result of corneal cross-linking by isolating an approximately 100 MHz increase in frequency shift following a cross-linking procedure purely due to changes of stromal tissue mechanics.

**Conclusions:**

Brillouin microscopy is sensitive to mechanical anisotropy of the stroma even in highly hydrated corneas. The agreement between model and experimental data suggested a quantitative relationship between Brillouin frequency shift, hydration state of the cornea, and stromal tissue stiffness.

**Translational Relevance:**

The protocol and model validated throughout this study offer a path for comprehensive measurements of corneal mechanics within the clinic; allowing for improved evaluation of the long-term mechanical efficacy of cross-linking procedures.

## Introduction

Corneal mechanical strength is primarily provided by the stromal layer, which comprises 90% of total corneal thickness. The corneal stroma consists of nanometer-thick collagen fibrils packed into 250 to 300 distinct fibers, or lamellae.^1^ Much effort has gone into understanding the underlying network of the collagen fibers, as the organization of the collagen network of the corneal stroma directly determines its biomechanical properties.^2^ The primary source of biomechanical strength is in the anterior third of the corneal stroma, where the collagen fibers interweave with one another.^3^^,^^4^ Severing the anterior fibers during the creation of a laser-assisted in situ keratomileusis (LASIK) flap can lead to the onset of cornea ectasia due to the overall decrease in mechanical properties^5^^,^^6^; and corneal cross-linking (CXL) has shown to increase the overall corneal strength by promoting the fiber-to-fiber connections primarily in the anterior part of the corneal stroma.^7^^,^^8^

From a mechanical standpoint, the cornea is generally approximated as a transversely isotropic material,^9^ meaning its mechanical properties are uniform on a given en face plane of the cornea but change if probed normal or perpendicular to the surface.^10^ X-ray diffraction^11^ and second/third harmonic generation^12^^,^^13^ studies have demonstrated that fibers display a strongly preferred direction parallel to the corneal surface, with only a small variation of ± 3.5°, and are stacked orthogonally layer by layer.^14^ The resulting mechanical anisotropy is a defining characteristic of the cornea: previous ex vivo measurements have reported the corneal stiffness to be hundred-fold larger when probed tangentially to the corneal surface (i.e. parallel to the collagen fibers) than when probed orthogonally to the corneal surface.^15^^–^^18^ Therefore, the corneal stiffness probed in a given direction will result, to first approximation, from combination of the tangential and perpendicular moduli in orthogonal directions. Cornea mechanics as a whole is also affected by the hydration state of the cornea,^19^^–^^21^ an important factor to consider given that the hydration state changes in both the short and long-term following clinical procedures.^22^^–^^24^

For the mechanical characterization of the cornea, Brillouin microscopy has recently emerged as an intriguing modality to perform measurements in vivo, in three dimensions, and without contact.^25^^–^^27^ Historically, measuring the Brillouin frequency shift has proved beneficial in studying the mechanical changes of the cornea, including characterizing cross-linking efficiency^28^^,^^29^ and monitoring the progression of keratoconus.^30^ Recently, the effects of water content on frequency shift have been investigated,^31^ and Brillouin microscopy has been used to detect the changes in corneal hydration that accompany Fuchs Corneal Dystrophy.^32^ From a mechanical standpoint, Brillouin microscopy probes micron-level unit volumes. In this scale, Brillouin microscopy measurements of anisotropy are not sensitive to macro-level changes in hydration unless such changes in hydration affect fiber distribution at a micron-scale level.^33^ Hereby, we will refer to such hydration-independent changes in mechanical properties as changes in solid mechanics or, equivalently, changes to stromal tissue.

Brillouin microscopy has not yet been demonstrated to detect the mechanical anisotropy of the cornea. Besides the practical consequence of an incomplete characterization of corneal mechanical properties, this has generated an important question as to whether, at a fundamental level, the Brillouin scattering phenomenon can be sensitive to mechanical properties that are independent to hydration at a micron-scale. The purpose of this study was to determine and demonstrate the sensitivity of Brillouin measurements to corneal mechanical anisotropy and isolate the contributions of hydration and solid mechanics on Brillouin frequency shift.

## Methods

### Brillouin Microscopy

A Brillouin microscopy setup similar to that previously described^34^ was used to image samples throughout this study. Briefly, a 660 nm laser with power of 15 mW was focused into the sample by an objective lens (20X/0.4 NA, Olympus Corporation of the Americas, Center Valley, PA) with lateral resolution of approximately 1 µm and depth resolution of approximately 4 µm. The scattered light, collected through the same objective, was coupled into a single mode fiber and delivered to a two-stage virtually imaged-phase-array (VIPA) spectrometer featuring an electron multiplying charge coupled device (EMCCD) camera (IXon Du-897; Andor Technology, Belfast, UK). Each Brillouin spectrum was acquired in 0.2 seconds. To quantify the Brillouin shift at each sample location, raw spectra from the camera were fitted using a Lorentzian function and calibrated using the known frequency shifts of water and methanol.

From the Brillouin frequency shift, the local longitudinal modulus of elasticity (*M’*) of the cornea can be estimated using the following relationship:

(1) $$M^{'}=\frac{\rho \Omega^{2}{\lambda_{i}}^{2}}{4n^{2}}$$

where Ω is the measured Brillouin frequency shift, *n* is the refractive index of the material, λ_*i*_ is the wavelength of the incident photons, and ρ is the density of the material. The spatially varying ratio of $\frac{\rho }{n^{2}}$ was approximated to the constant value of 0.57 g/cm^3^ based on literature values^35^^–^^39^; we estimate this to introduce a 0.3% uncertainty throughout the cornea. We have previously validated the strong correlation (R^2^ = 0.98) between changes in Brillouin-derived longitudinal modulus of elasticity and tangent modulus of porcine corneas at very low strain (< 10%).^29^

### Measurements of Porcine Cornea Globes and Buttons

Porcine eyes were obtained on the day of enucleation from a local slaughterhouse (Wagner Meats, Mount Airy, MD) as well as the day following enucleation via overnight delivery (J&J Packing Company, Brookshire, TX); all eyes were kept on ice until the start of each experiment. All corneas were inspected prior to use and damaged or unclear tissue was discarded prior to experimentation. For all eyes, the epithelium was removed by careful debridement with a razor blade. Eyes were randomly assigned to either be measured as intact globes or first dissected in order to measure central 5.0 mm punched buttons (Disposable Biopsy Punch; Integra Miltex, York, PA).

**Figure 1.**
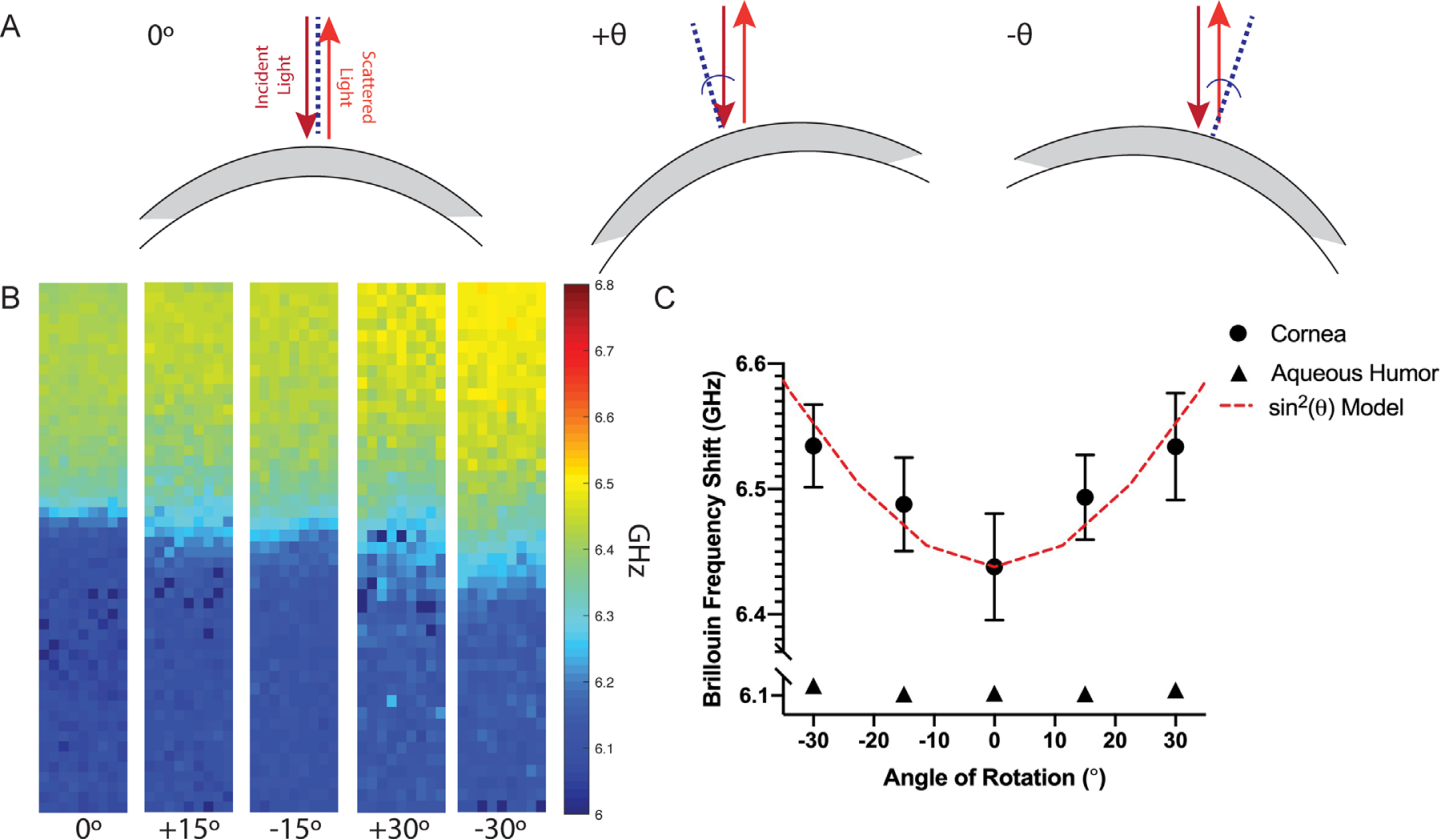
(**A**) Schematic demonstrating the probing angle of the incident and collected scattered light relative to the corneal surface. The 0° indicates the incident laser is positioned orthogonal to the corneal surface; +/− indicates the directionality relative to 0°. The *dotted blue line* shows the normal path to the corneal apex. (**B**) Representative Brillouin frequency maps (200 µm × 1200 µm) of a single cornea scanned at 0°, +15°, −15°, +30°, and −30° inclination relative to the incident laser. The globes are depicted anterior upward with aqueous humor at the bottom of the image. The warmer colors correspond to a higher frequency shift. (**C**) Average Brillouin frequency shift of the anterior third of (*n* = 10) identical corneas (*dots*) and a 200 µm × 100 µm sample of aqueous humor (*triangles*) measured at 0°, +15°, −15°, +30°, and −30° inclination relative to the incident laser. A sin^2^ model (*dotted line*) was plotted with a wavelength of 180° fit to the experimental data. Error bars represent the standard error of the mean for each inclination and are within the size of the symbol when not seen.

Both cornea globes and buttons were mounted on the Brillouin microscope using a 360° inclinational stage with 1° measurable increments. Prior to mounting on the stage, button samples were rested on a microscope slide via a coupling gel (Systane Ultra Lubricating Eye Drops). As demonstrated by Figure 1A, the samples were measured at a selected angle respective to the incident laser with 0° indicating the incident laser was positioned orthogonal to the corneal surface and +/− indicating inclination directionality. Based on this directional axis, a 90° rotation would position the incident laser parallel to the preferred fiber direction; thereby, tangential to the corneal surface. For the intact globes, scanning order was 0°, +15°, −15°, +30°, and −30°. For the buttons, scanning order was 0° followed by 30°. Each scan, including the time it took to rotate the cornea and reposition the incident beam, was no longer than 3.5 minutes. The number of angle variations was chosen to minimize the total time between the first and last scan in order to limit dehydration artifacts while still demonstrating an evident difference in probing direction. The maximum angle of 30° was chosen in order to maintain adequate signal intensity of our setup and to minimize optical artifacts from tilting the sample. It is worth noting that with each inclination, the corneal thickness appears to increase in the Brillouin image as it is probed at an angle. For analysis, the thicknesses of the scans were normalized and the anterior third of the cornea was used for analysis while a sample (100 µm × 200 µm) of aqueous humor within the eye globe or lubricant gel applied to the posterior of the cornea button was used as respective, isotropic controls.

### Corneal Hydration/Dehydration

Corneal hydration was controlled and monitored throughout the experiments. Prior to a scan, corneal buttons were subjected to hydration/dehydration protocols similar to Shao et al*.*^31^ in which the button was immersed in distilled water for 30 to 180 minutes to induce hydration or left out in air for 15 to 30 minutes to induce dehydration. Following each respective protocol, the cornea was weighed (*W’*) and imaged via Brillouin microscopy. The cornea was then left out to completely dry in air for 72 hours and reweighed (*W_0_*). The hydration (*H*), a unitless ratio between the mass of the water content and dry mass of the cornea, at the time of each measurement was then calculated as:

(2) $$H=\frac{W^{'}-W_{0}}{W_{0}}$$

### Corneal Cross-Linking 

CXL was used to vary the mechanical properties of corneal buttons. One drop of riboflavin solution (riboflavin 0.1% and dextran 10%) was topically applied to the button every 3 minutes for 20 minutes. Then, the saturated button was exposed to a constant 9 mW/cm^2^, 365 nm UV-A light (UV Curing LED System; Thorlabs, Newton, NJ) for 10 minutes, during which one drop of riboflavin solution was administered at the 5-minute interval. An accelerated cross-linking protocol was used in order to minimize any variation in tissue hydration and expedite overall procedure time.

### Modeling Brillouin Shift Dependence on Hydration and Solid Mechanics

Previously, Shao et al. described the dependence of corneal Brillouin shift from corneal hydration.^31^ We can extend the aforementioned model to include the effect of solid mechanics. The Brillouin frequency shift is a function of incident wavelength (λ), longitudinal modulus of elasticity (*M*), mass density (ρ), and refractive index (*n*) as shown by:

(3) $$\Omega =\frac{2}{\lambda }n\sqrt{\frac{M}{\rho }}=\;\frac{2}{\lambda }n\sqrt{\frac{1}{\rho \beta }}$$

Given that, for soft tissues, one can approximate the Poisson's ratio as approximately 0.5 and, therefore, nearly incompressible, bulk modulus is dramatically higher than shear modulus. Therefore, the bulk modulus is approximately equal to the longitudinal modulus of elasticity. Thus, tissue compressibility (β), which normally is considered as the inverse of the bulk modulus, can be approximated as the inverse of the longitudinal modulus of elasticity.^40^ This approximation follows the model of Shao et al.^31^ To account for the changes in Brillouin shift due to hydration, we can use the following biphasic approximation as performed in the original Brillouin shift model:

(4a) $$n=n_{w}f_{w}+n_{s}\left(1-f_{w}\right)$$

(4b) $$\rho =\rho_{w}f_{w}+\rho_{s}\left(1-f_{w}\right)$$

(4c) $$\beta =\beta_{w}f_{w}+\beta_{s}\left(1-f_{w}\right)$$

The subscripts *_w_* and *_s_* denote the contributions from the water and solid components respectively; *f_w_* denotes the volume fraction of water within the tissue.

Following similar notation as Shao et al., we can indicate the changes in volume fraction of water as:

(5) $$f_{w}=f_{w0}\left(1+x\right)$$

where the subscript _0_ implies a physiological value; and the variable $x=\frac{H-H_{0}}{4H+H_{0}}$ conveniently describes the change in tissue hydration *H* as defined in Equation 2. This allows Equations 4a,b,c to be rewritten in terms of the hydration change x:

(6a) $$\Delta n=\left(n_{w}-n_{s}\right)f_{w0}x\;$$

(6b) $$\Delta \rho =\left(\rho_{w}-\rho_{s}\right)f_{w0}x$$

(6c) $$\Delta \beta =\left(\beta_{w}-\beta_{s}\right)f_{w0}x$$

The model, thus far, only describes changes in the quantities due to hydration. However, whereas index and density changes can be fully described by changes in hydration, the compressibility of the sample can also change as a result of changes in solid mechanics independent of hydration. Thus, the compressibility in Equation 6c needs to include an additional term reflecting changes in solid compressibility (Δβ_*s*_):

(7) $$\Delta \beta =\left(\beta_{w}-\beta_{s}\right)f_{w0}x+\Delta \beta_{s}\left(1-f_{w0}\right)$$

We can, therefore, derive an equation to describe the Brillouin frequency shift behavior as a result of small changes in both hydration and solid mechanics:

(8) $$\begin{array}{c} \Omega =\;\frac{2}{\lambda }\left(n_{0}+\Delta n\right)\sqrt{\frac{1}{\rho_{0}+\Delta \rho }}\sqrt{\frac{1}{\beta_{0}+\Delta \beta }}\\ =\Omega_{0}\times \left(1-0.114x\right)\times \sqrt{1-0.19x}\times \\ \sqrt{1+0.77x+5.54\times 10^{8}\Delta \beta_{s}} \end{array}$$

where all values for numerical parameters are the same as Shao et al.^30^

### Statistical Analysis

To compare the Brillouin-derived longitudinal modulus of elasticity of corneal buttons rotated at 0° and 30° as well as the Brillouin frequency shift of 0° repeated measurements, a Wilcoxon Signed-Rank Test was performed. To compare the Brillouin frequency shift of virgin and cross-linked corneas, a Wilcoxon Rank Sum Test was performed. For measurement consistency, throughout the paper we reported average values ± standard error of anterior third of the stroma.

## Results

### Mechanical Anisotropy of Intact Porcine Corneas

We observed a dependence of Brillouin frequency shift on the angle of inclination of the cornea (see Fig. 1). Intact corneas (*n* = 10) were mounted on an inclinational stage and imaged via Brillouin microscopy at 0°, +15°, −15°, +30°, and −30° relative to the incident laser. Figure 1B depicts representative Brillouin maps of a single cornea scanned at 0°, +15°, −15°, +30°, and −30°. As indicated by the warmer colors, the Brillouin frequency shift increases with the angle of inclination and symmetrically with respect to the 0° configuration (i.e. the configuration where the probed modulus is perpendicular to the direction of the collagen fibers). Figure 1C shows a quantification of this effect for *n* = 10 intact corneal samples. The frequency shift was at a minimum (6.44 ± 0.04 GHz) when the cornea was rotated 0° to the incident laser (i.e. when the measurements were probed orthogonal to the collagen fiber direction). As the cornea was rotated further from the initial 0° position, the measured shift followed a sinusoidal-squared relationship with probing angle with an average frequency shift of 6.49 ± 0.03 GHz, 6.49 ± 0.04 GHz, 6.53 ± 0.03 GHz, and 6.53 ± 0.03 GHz at +15°, −15°, +30°, and −30°, respectively. The average shifts of equal but opposite angles (i.e. +15° and −15°) displayed the same frequency shift within the instrument sensitivity, demonstrating hydration effects due to dehydration were negligible. Further evidence that the changes in frequency shift were due to mechanical anisotropy was the constant behavior of aqueous humor within each eye globe, which was used as an isotropic control. From the experimental start to finish (0° vs. −30°), the average frequency shift of aqueous humor deviated only slightly (from 6.11 ± 0.02 GHz to 6.13 ± 0.01 GHz) and was ascribed to a loss of signal strength in the posterior portion of the sample and/or instrument sensitivity error.

### Mechanical Anisotropy of Corneal Buttons

**Figure 2.**
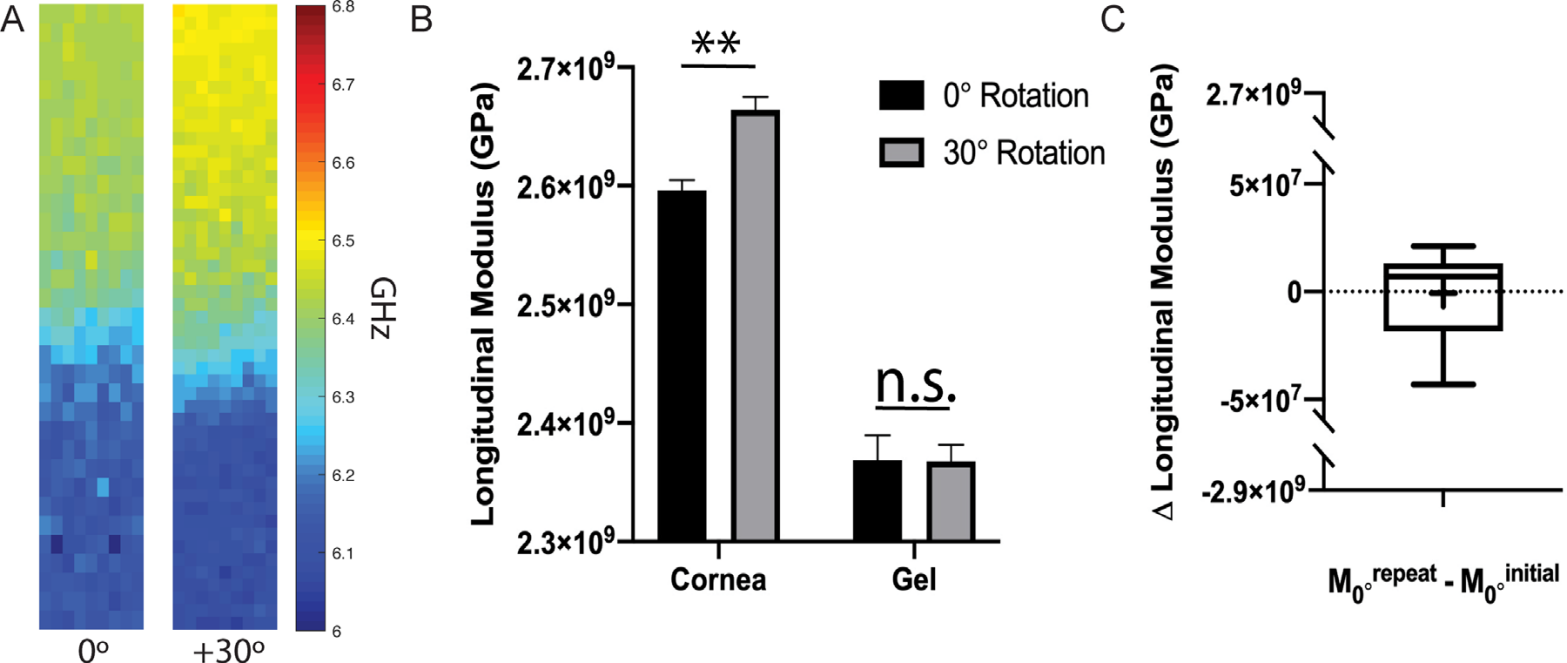
(**A**) Representative Brillouin frequency maps (200 µm × 1200 µm) of a single cornea and gel scanned at 0° and 30°. The cornea is positioned anterior upward. The warmer colors correspond to a higher frequency shift and, thereby, a higher longitudinal modulus of elasticity. (**B**) Average longitudinal modulus of elasticity derived via Brillouin frequency shift, of (*n* = 10) individual corneal anterior sections as well as the isotropic gel applied to the posterior of each sample rotated at 0° and 30°. Error bars represent standard error of the mean for each condition. (**C**) Difference in Brillouin-derived longitudinal modulus of elasticity between (*n* = 10) identical, repeated measurements of corneas scanned at 0°. The cross within the box represents the mean value while the horizontal line represents the median. (** = *P* < 0.01).

To increase the control over the measurement conditions, experiments were performed on central corneal buttons punched from ex vivo porcine globes. Figure 2A shows representative images of the same cornea sample measured at 0° and 30°. The measured Brillouin frequency shifts were converted to longitudinal modulus of elasticity using Equation 1, using the incident laser wavelength of 660 nm and assuming a constant index of refraction and density of 1.37 and density of 1080 kg/m^3^ for corneal tissue.^26^^,^^31^ Figure 2B demonstrates the difference in longitudinal modulus of elasticity, derived via Brillouin microscopy, between identical corneas (*n* = 10) measured at 0° then 30°. The average longitudinal modulus of elasticity ± standard error of the anterior third section of the corneas at +30° inclination (2.66 ± 0.03 GPa) significantly differed (*P* < 0.01) from the same corneas measured at 0° inclination (2.60 ± 0.03 GPa). As an isotropic sample control, index-matching, lubricant gel was placed on the posterior side of the cornea and measured at each inclination. The gel at 30° and 0°, 2.37 ± 0.06 GPa and 2.37 ± 0.04 GPa, respectively, did not significantly differ (*P* > 0.05) and the difference between the two was within instrument sensitivity (10 MHz). To test for unwanted changes in Brillouin-derived longitudinal modulus of elasticity due to dehydration and/or temperature changes between scans, (*n* = 10) corneas were measured at 0° (M_0°_^initial^) followed by a repeated measurement again at 0° (M_0°_^repeat^); replicating the time between measurements at 0° then 30°. As shown in Figure 2C, the average difference in longitudinal modulus of elasticity between the repeated measurements (M_0°_^repeat^ - M_0°_^initial^) was not significantly different than zero (*P* > 0.05) and within the instrument sensitivity, indicating no change in frequency shift due to the time between scans.

### Mechanical Anisotropy at Varying Hydration Levels

**Figure 3.**
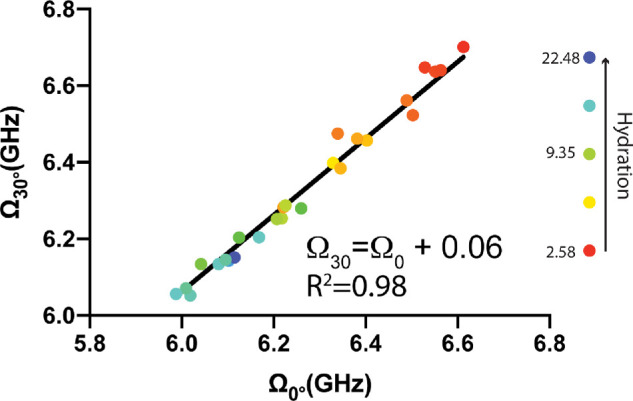
Average Brillouin frequency shifts of the anterior third of individual corneas (*n* = 26) imaged at 0° (Ω_0°_) and 30° (Ω_30°_). The samples varied in hydration from 2.58 to 22.49, as the colder and warmer colored dots correspond to higher and lower levels of hydration respectively. A line with a slope fixed at 1.0 was fit as $\Omega_{30^{\circ }}=\;\Omega_{0^{\circ }}+\;0.06$ (R^2^ = 0.98).

We next determined how the sensitivity to mechanical anisotropy of Brillouin microscopy was affected by the hydration level of the cornea samples. To do this, we compared the Brillouin frequency shift of (*n* =26) corneas, each with a distinct level of hydration resulting from a previously described dehydration / hydration protocol, measured at 0° (Ω_0°_) and then 30° (Ω_30°_). The average frequency shift of the anterior third of each cornea was calculated at both inclinations and Ω_30°_ vs. Ω_0°_ was plotted (Fig. 3). The data within Figure 3 includes a large range of corneal hydrations throughout the experiment from 2.58 to 22.49, corresponding to 72.0% and 95.7% water content, respectively. The water content of most species is approximately 76% in normal physiological conditions, which corresponds to a hydration level of 3.2 (=H_0_) ^41^; therefore, our range included all values within physiological expectation. Experimental data are fit extremely well by the linear fit $\Omega_{30^{\circ }}=\;\Omega_{0^{\circ }}\;+\;0.06$ (R^2^ = 0.98) with slope 1.0 and origin intercept of 60 MHz.

### Decoupling Brillouin Shift Dependence on Hydration and Solid Mechanics in CXL Procedures

**Figure 4.**
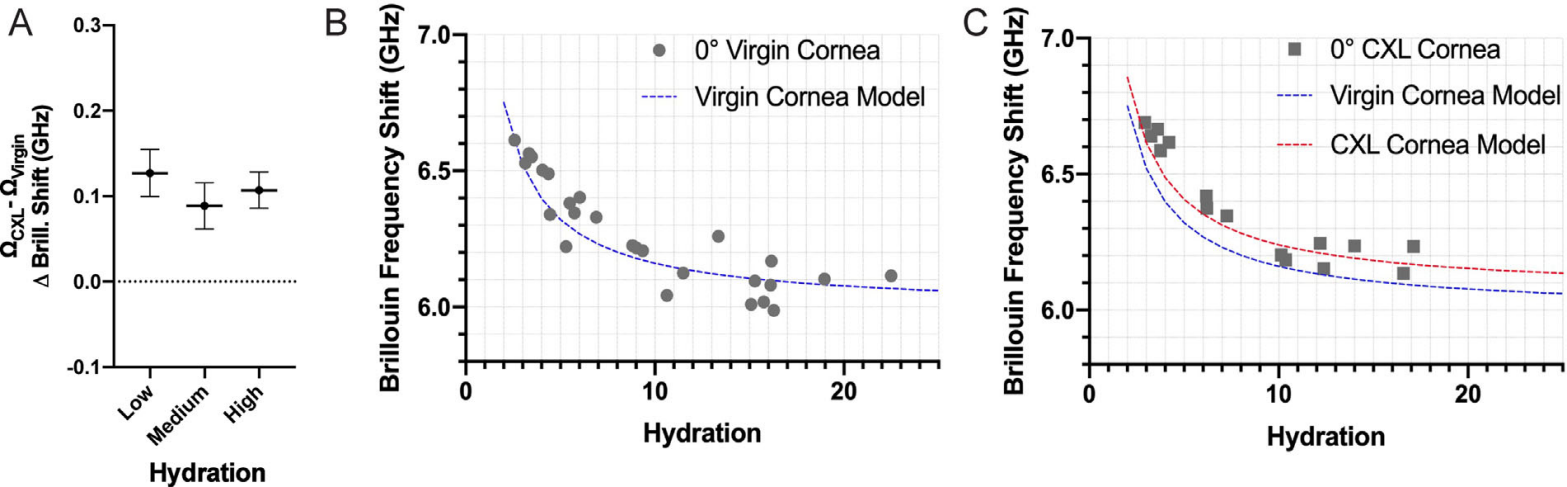
(**A**) Difference in average Brillouin frequency shifts between CXL and virgin corneal buttons within varying hydration groupings: low (H < 5), medium (5 < H < 10), and high (H > 10) hydration. The *horizontal line* and error bars represent the mean of each grouping and standard error of the mean respectively. (**B**) Average Brillouin frequency shift of the anterior of (*n* = 26) virgin corneas versus the measured hydration (*dots*). Computational model of the relationship between Brillouin frequency shift, hydration, and solid compressibility in which Δβ_*s*_ = 0 (*blue line*). (**C**) Average Brillouin frequency shift of the anterior of (*n* = 15) cross-linked corneas versus the measured hydration (*squares*). Computational model of the relationship among Brillouin frequency shift, hydration, and solid compressibility in which Δβ_*s*_ = 5.3*10^−11^ Pa^−1^ (*red line*). The Virgin model in which Δβ_*s*_ = 0 was identical represented (*blue line*).

It is widely expected that CXL affects corneal mechanics both via immediate hydration changes and via more lasting changes in solid mechanics.^22^^,^^42^^,^^43^ Corneal buttons in virgin conditions (*n* = 26) and CXL conditions (*n* = 15) of varying hydrations (from 2.58 to 22.49) were prepared and measured according to the procedures described in the methods section. A statistically significant difference was measured between virgin and cross-linked corneas at low, medium, and high hydration (Table). Furthermore, the difference between cross-linked and virgin groups remained nearly constant at all levels of hydration (Fig. 4A).

**Table.** Brillouin Frequency Shift of Virgin versus CXL Corneas

Hydration	Ω_virgin_ (GHz)	Ω_CXL_ (GHz)	Significance
Low (H < 5)	6.51 ± 0.03	6.64 ± 0.02	** (*P* < 0.01)
Medium (5 < H < 10)	6.29 ± 0.03	6.38 ± 0.02	* (*P* < 0.05)
High (H > 10)	6.09 ± 0.02	6.20 ± 0.02	** (*P* < 0.01)

To further understand the dependence on hydration and solid mechanics, the Brillouin frequency shift of the anterior section of each cornea was measured and plotted as a function of hydration (Fig. 4B). Figure 4B also shows the prediction of Equation 8 (*blue line*) as the theoretical model describing the dependence of Brillouin frequency shift on hydration. Here, Ω_0_ was fit to 6.49 GHz and Δβ_*s*_ constrained to 0 Pa^−1^ as we assumed no changes in solid compressibility due to changes in hydration. We then plotted Brillouin shifts of virgin and CXL corneas as a function of hydration in the same plot (Fig. 4C). Here, Equation 8 was plotted (*red line*) with an identical Ω_0_ of 6.49 GHz, whereas Δβ_*s*_ was fit to the cross-linked data as a free parameter and best fit resulted to be 5.3 × 10^−11^ Pa^−1^. It can be clearly seen that the experimental data follows the model prediction well in both virgin and CXL conditions (i.e. the Brillouin shift changes as a function of hydration are consistent with the model in both conditions). Importantly, the CXL corneas have distinctly higher Brillouin shifts purely due to the mechanical response of the solid component(s) Δβ_*s*_ independent of hydration. Specifically, the change in Brillouin shift due purely to CXL-induced solid mechanics modifications amount to approximately 100 MHz, which can be easily measured by current Brillouin clinical instruments.

## Discussion

The anisotropy of the cornea is central to the mechanical function of the cornea and comes from the unique architecture of collagen fibers within the stroma: the Young's modulus probed parallel to the collagen fiber orientation has been shown to be remarkably larger than the one probed orthogonal to the fiber direction.^15^^–^^18^ Brillouin microscopy is an emerging modality capable of probing corneal mechanics without contact or perturbations, thus offering unique in vivo potential. However, until now, no Brillouin measurement has detected the mechanical anisotropy present within a cornea.

Brillouin microscopy measures the frequency shift induced by light interacting with acoustic phonons propagating within a material; in back-scattering configuration, it probes the high-frequency longitudinal modulus of elasticity of a material in the direction of the incident laser. Previously, Brillouin microscopy had been shown to detect mechanical anisotropy of nonbiological samples or isolated fibers^44^^–^^46^ but not of the cornea, or other biological tissues. Naturally, this has raised the question as to whether the Brillouin phenomenon in biological, hydrated samples loses sensitivity to mechanical properties that are intrinsic to the solid collagen network of the stromal tissue. In order to evaluate the sensitivity of Brillouin microscopy to mechanical anisotropy, cornea samples were imaged at varying angles of inclination and various levels of hydration. Because Brillouin measurements probe the longitudinal modulus of elasticity along the direction of the laser beam in back-scattering geometry, as we probed higher angles of corneal inclination (i.e. closer to parallel with the collagen fibers), the Brillouin frequency shift increased. Due to the relationship between frequency shift and longitudinal modulus of elasticity, we can deduce that Brillouin microscopy detected higher mechanical modulus as the inclination of the collagen fibers within the cornea changed from orthogonal to parallel to the incident laser. Specifically, the increase followed the expected sin^2^(θ) relationship consistent with expectations and previous literature for a material with transverse mechanical anisotropy with two principal moduli along perpendicular and parallel directions.^45^^,^^46^ Importantly, as demonstrated by the constant slope of one in Figure 3, the mechanical anisotropy detected between 0° and 30° remained constant throughout hydration conditions ranging from 72.0% and 95.7% water content. The 60 MHz origin intercept is consistent with Figure 2 and can be interpreted as the frequency shift due solely to the solid mechanical differences, independent from hydration, between a cornea measured at 30° and 0°. This suggests that, unlike synthetic hydrogels where Brillouin microscopy was found to lose sensitivity to mechanical changes at high water content,^40^^,^^47^ Brillouin microscopy does not lose sensitivity to changes in solid mechanics of the cornea even in highly hydrated samples.

The ability to detect mechanical anisotropy also enabled us to isolate the contributions of hydration and solid mechanics on Brillouin frequency shift. Previously, Shao et al. evaluated the relationship between hydration and Brillouin frequency shift by quantifying the small changes in refractive index, density, and compressibility due to changes in water content of a cornea.^31^ However, several procedures (e.g. corneal cross-linking) can also affect the solid constituents of the cornea, including collagen network and extracellular matrix, independent from hydration; thus the corresponding changes in modulus and Brillouin shift need to be quantified.^48^ Therefore, although the hydration dependence fully and accurately describes changes in refractive index and density, the change in compressibility should be sensitive to changes in both hydration and solid compressibility. To account for such changes, we extended the model by Shao et al.^31^ to include changes in compressibility that were independent from hydration. Doing so resulted in a quantitative model relating Brillouin frequency shift to both hydration and solid mechanics.

To test our model, we measured the frequency shift of virgin and cross-linked corneas at a variety of hydration conditions. By controlling the hydration, we were able to isolate the differences in frequency shift between the virgin and cross-linked samples that were entirely due to the changes in solid mechanics of the stromal tissue. These changes correspond to an average Brillouin frequency increase of 100 MHz, between virgin and cross-linked corneas at constant hydration, which is easily measurable with current clinical Brillouin instruments. Based on our previous correlation between changes in Young's Modulus and Brillouin modulus,^29^ a 100 MHz increase in frequency shift would suggest an approximately 25% increase in Young's Modulus following CXL due solely to changes in solid mechanics independent from hydration. However, given the improved understanding provided from our model and experimental data, future studies will be needed for a quantitative relationship between Young's and Brillouin-derived modulus. Our results suggest that CXL corneal stiffening needs to be evaluated more thoroughly. Cross-linking has been shown to increase the corneal modulus, via Brillouin microscopy^8^^,^^26^ and other mechanical testing methods,^23^^,^^49^^–^^51^ by altering the solid mechanics and, on a shorter timescale, the water content. In vivo, due to the riboflavin / dextran solution used, the corneal thickness is decreased during the procedure due to dehydration and recovers to its pre-operative hydration state days to weeks following re-epitheliazation.^22^ Additionally, the mechanical stiffening effects immediately measured after cross-linking have been reported to diminish over a similar timetable following the procedure.^52^^–^^55^ This would suggest that the immediate, large stiffening effect measured ex vivo by many papers following the cross-linking procedure could largely be due to the dehydration experienced throughout the procedure. In this respect, our results provide a protocol to discriminate the mechanical effects due to hydration versus the solid components. In the future, we will perform a quantitative calibration between Brillouin measurements and gold-standard stress-strain tests. Then, by using Brillouin measurements and hydration via thickness measurements, our protocol and model are expected to be useful in the clinic to determine the long-term mechanical efficacy of cross-linking procedures.

There are limitations to our study. The current study provides information on the transverse corneal anisotropy (i.e. when the cornea is rotated in respects to the depth axis). However, there are a number of benefits to assessing in-plane anisotropy by comparing the stiffness across the nasal-temporal direction versus the superior-inferior direction.^29^ In the future, now that we have proven Brillouin microscopy is capable of measuring corneal anisotropy, we will look to measure mechanical differences across other directions within the cornea. Additionally, the procedure performed in this study refers to the central and anterior part of the porcine cornea (0.2 × approximately 0.25 mm). Therefore, the mechanical anisotropy will need to be characterized in the human cornea to gain specific information of clinical procedures on middle or peripheral sections of the human cornea with potentially different orientation of collagen fibers. Furthermore, in our ex vivo experiments, we measured corneal weight to quantify hydration; to practically translate these procedures to clinical setting, hydration will need to be quantified via corneal thickness.

In conclusion, by combining Brillouin measurements of different geometrical configuration, we demonstrated that Brillouin measurements are sensitive to the mechanical anisotropy of the cornea. For Brillouin measurements performed on intact eye globes, both in vivo and ex vivo, this sensitivity must be taken into account as the frequency shift will be dependent on the angle of the collagen fibers, thereby, the natural curvature of the eye. Therefore, similar to the recently developed polarization-sensitive (PS)- optical coherence tomography (OCT), which compensates for scanning angle-induced artifacts,^56^^,^^57^ en face Brillouin maps of the cornea can be normalized based on the angle dependence of Brillouin shift found in this study. This ability to detect mechanical anisotropy also enabled us to isolate the contributions of hydration and solid mechanics on Brillouin frequency shift. This is expected to pave the way toward obtaining a quantitative relationship between Brillouin measurements and gold-standard mechanical tests offering a path for noninvasive and comprehensive measurements of corneal mechanics in vivo.
